# Enhancement of Cisplatin-Mediated Apoptosis in Ovarian Cancer Cells through Potentiating G2/M Arrest and p21 Upregulation by Combinatorial Epigallocatechin Gallate and Sulforaphane

**DOI:** 10.1155/2013/872957

**Published:** 2013-02-17

**Authors:** Huaping Chen, Charles N. Landen, Yuanyuan Li, Ronald D. Alvarez, Trygve O. Tollefsbol

**Affiliations:** ^1^Department of Biology, University of Alabama at Birmingham, CH175, 1300 University Boulevard, Birmingham, AL 35294-1170, USA; ^2^Department of Obstetrics and Gynecology, University of Alabama at Birmingham, Birmingham, AL 35294, USA; ^3^Center for Aging, University of Alabama at Birmingham, Birmingham, AL 35294, USA; ^4^Comprehensive Cancer Center, University of Alabama at Birmingham, Birmingham, AL 35294, USA; ^5^Nutrition Obesity Research Center, University of Alabama at Birmingham, Birmingham, AL 35294, USA; ^6^Comprehensive Diabetes Center, University of Alabama at Birmingham, Birmingham, AL 35294, USA

## Abstract

Advanced-stage ovarian cancer is characterized by high mortality due to development of resistance to conventional chemotherapy. Novel compounds that can enhance the efficacy of conventional chemotherapy in ovarian cancer may overcome this drug resistance. Consumption of green tea (epigallocatechin gallate, EGCG) and cruciferous vegetables (sulforaphane, SFN) is inversely associated with occurrence of ovarian cancer and has anticancer effects through targeting multiple molecules in cancer cells. However, the effects of EGCG and SFN combinational treatment on ovarian cancer cells and on efficacy of cisplatin to these cells are unknown. In this study, EGCG or SFN was used to treat both cisplatin-sensitive (A2780) and cisplatin-resistant (A2780/CP20) ovarian cancer cells alone or in combination with cisplatin. We found that EGCG and SFN combinational treatment can reduce cell viability of both ovarian cancer cell lines time- and dose-dependently. Furthermore, EGCG and SFN combinational treatment can enhance cisplatin-induced apoptosis and G2/M phase arrest, thereby enhancing the efficacy of cisplatin on both cisplatin-sensitive and cisplatin-resistant ovarian cancer cells. EGCG and SFN combinational treatment upregulated p21 expression induced by cisplatin in cisplatin-sensitive ovarian cancer cells, while p27 expression was not regulated by these treatments. Collectively, these studies provide novel approaches to overcoming cisplatin chemotherapy resistance in ovarian cancer.

## 1. Introduction

Ovarian cancer has the highest mortality among gynecologic cancers. Most patients with ovarian cancer are diagnosed at late stages due to lack of effective screening strategies and specific symptoms associated with early-stage disease. Conventional treatment for late stages of ovarian cancers is surgical excision followed by platinum/taxane combination chemotherapy. Although this treatment regime is effective as the first-line treatment, recurrence occurs in up to 75% of ovarian cancer patients. Patients with recurrent ovarian cancer ultimately develop resistance to chemotherapy and eventually succumb to the disease [1].

Thus, drug resistance is an urgent problem in the current treatment for ovarian cancer. The inefficiency of current conventional chemotherapies to kill cancer cells in a timely manner, which can allow necessary time for ovarian cancer cells to evolve drug resistance under pressure of selection, has been theorized to be one reason chemotherapy resistance develops [2]. Therefore, novel therapies are needed which can improve the efficacy of conventional chemotherapy in drug-sensitive ovarian cancer cells in order to greatly minimize the chance of drug resistance or that can improve the response to drugs in resistant ovarian cancer cells. In this regard, combination treatment with different compounds that target several different pathways has appeared as a promising direction for overcoming the drug resistance problem [3].

Epidemiological studies have revealed an inverse association between the dietary intake of green tea, cruciferous vegetables such as broccoli, Brussels sprouts, or cabbage, and the occurrence of certain types of cancers, including ovarian cancer [4–7]. The anticancer effects of the major active component of green tea, epigallocatechin gallate (EGCG), or of broccoli, sulforaphane (SFN), have thus received considerable attention in recent years. EGCG has been shown to induce apoptosis in breast cancer cells [8] to target cancer cells through a variety of mechanisms, including decreasing expression of ET-1 and its receptor ETAR (which are also overexpressed in ovarian cancer cells) [9] and reducing expression of *hTERT*, the major catalytic subunit of telomerase [10, 11]. Furthermore, EGCG has been identified as a DNA methyltransferase (DNMT) inhibitor in various cancer types [12]. SFN has been shown to exert its anticancer effects through Nrf2-mediated induction of phase 2 detoxification enzymes, thus elevating the cell defense against oxidative damage and promoting the removal of carcinogens [13]. SFN has also been observed to suppress cytochrome P450 enzymes [14], suppress cell cycle progression [15], induce apoptotic pathways [15], and inhibit angiogenesis and inflammatory response [16, 17]. More recently, SFN has been shown to modulate the epigenetic status of genes involved in cancer cell survival pathways via inhibiting histone deacetylase (HDAC) activity [18].

As aforementioned, EGCG and SFN can inhibit cancer cells by impacting largely different molecular targets; combinational treatment with these agents thus has the potential to exert a stronger cancer inhibition effect than administering either single compound alone. It would be important to determine whether the combinational treatment of these two compounds can exert a stronger anticancer effect or can enhance the efficacy of conventional chemotherapy. Additionally, determining the effects and mechanisms of EGCG and SFN on ovarian cancer cells at physiological doses of EGCG or SFN will provide critical knowledge for developing potential novel treatments for ovarian cancer [19].

The present study was thus performed to determine the effect of EGCG and SFN combinational treatment on both cisplatin-sensitive and cisplatin-resistant ovarian cancer cells and the enhancement of cisplatin efficacy on these cancer cells and to elucidate the potential mechanisms responsible for the phenomenon. Our results indicate that SFN can inhibit both cisplatin-sensitive and cisplatin-resistant ovarian cancer cells while EGCG can enhance the inhibiting effect of SFN in both cell types. Furthermore, EGCG and SFN combination treatment can enhance the efficacy of cisplatin to both cisplatin-sensitive and cisplatin-resistant ovarian cancer cells. Mechanistic studies reveal that EGCG and SFN combination treatment can upregulate p21 expression, while EGCG, SFN, and cisplatin treatment can induce an even higher level of p21 expression compared with EGCG and SFN combinational treatment alone. These findings reveal for the first time that EGCG and SFN combination treatment can inhibit ovarian cancer cells by upregulating p21 which contributes to the enhanced efficacy of cisplatin in ovarian cancers. 

## 2. Materials and Methods

### 2.1. Cell Culture

A2780 and A2780/CP20 cells were grown in RPMI 1640 medium (Mediatech Inc, Manassas, VA, USA) supplemented with 10% fetal bovine serum (Atlanta Biologicals, Lawrenceville, GA, USA) and 1% penicillin/streptomycin (Mediatech, Herndon, VA, USA). Cells were maintained in a humidified environment of 5% CO_2_ and 95% air at 37°C. Ovarian cancer cells were treated with various concentrations of EGCG (Sigma, St. Louis, MO, USA) or R,S-sulforaphane (LKT Laboratories, Minneapolis, MN, USA) for the indicated time intervals. EGCG or R,S-sulforaphane was prepared in DMSO with a stock concentration of 100 mM/L at −20°C. The concentration of DMSO in medium was less than 0.1% (v/v). Cells treated with DMSO served as a vehicle control. Medium with fresh EGCG or SFN was changed every 24 h.

### 2.2. MTT Assay

The 3-(4,5-dimethylthiazol-2-yl)-2,5-diphenyltetrazolium bromide (MTT) assay was used to test the viability of ovarian cancer cells. Approximately 5000 ovarian cancer cells were seeded in each well in 96-well plates. Cells were treated as indicated after 24 h. At the end of each treatment, 10 *μ*L of 1 mg/mL MTT was added to each well and incubated for 2 h at 37°C. At the end of a 2 h incubation period, the medium was aspirated and 200 *μ*L DMSO was added to each well to dissolve the formazan crystals. Dye absorbance in each well was measured at 595 nm, with 630 nm as a reference wavelength. 

### 2.3. Analysis of Cell Cycle Progression

Propidium iodide (PI) staining-based flow cytometry cell cycle assay was used to analyze cell cycle distribution. Approximately 2 × 10^5^ cells were plated in each well of 6-well plates with a 2 mL volume of medium for each well. Medium containing freshly dissolved EGCG or SFN was added 24 h later and changed daily. Cells were harvested at the indicated time points from 6-well plates by trypsinization. After washing with PBS, they were fixed in 70% ethanol at −20°C overnight. Cells were then centrifuged and washed with PBS the second day. Cells were suspended in PBS containing 0.1% Triton X-100, 0.1% RNase, and 50 *μ*g/mL PI and incubated in dark for about 30 min. DNA contents in stained nuclei were then analyzed with flow cytometry. 

### 2.4. Analysis of Apoptosis

Annexin V and PI double-staining-based flow cytometry apoptosis assay was used to determine the effect of EGCG and SFN treatment on apoptosis. Cells were harvested with trypsinization followed by washing with PBS. Cells were then stained with Annexin V and PI (Invitrogen) in the binding buffer for 15 min in the dark. Cells were analyzed through flow cytometry.

### 2.5. Western Blotting

Western blotting was used to assess protein expression. Cells were washed with PBS and collected in RIPA lysis buffer with protein inhibitors (Upstate Biotechnology, Charlottesville, VA, USA). Cellular protein was extracted according to the manufacturer's protocol. Protein samples (20 *μ*g) were electrophoresed on 10% SDS-polyacrylamide gels and transferred to PDVF membranes. Antibodies against p21 (Millipore, CA, USA) and *β*-actin (cell signaling) were used to probe corresponding proteins. Immunoreactive bands were visualized using the enhanced chemiluminescence detection system (Santa Cruz Biotechnology) following the protocol of the manufacturer. Proteins were identified according to their size by comparing them to molecular weight markers run on the same gel.

### 2.6. Real-Time Quantitative PCR

Total cellular RNA was isolated using an RNeasy mini kit (Qiagen, Valencia, CA, USA) according to the manufacturer's instructions. Two micrograms of total RNA were reverse-transcribed into cDNA using the iScript cDNA synthesis kit (Bio-rad, Hercules, CA, USA). The PCR primer sets that were used are as the following: 5-TGTCCGTCAGAACCCATG-3 (F), 5-TGGGAAGGTAGAGCTTGG-3 (R) for *p21*, 5-CCACGAAGAGTTAACCCGGG-3 (F), 5-GTCTGCTCCACAGAACCGGC-3 (R) for *p27*, 5-GTTCTCCGGGAGATGTTGCATA-3 (F), 5-TGGTGGTGTTGAGAAGGTATAACTTG-3 (R) for *hMLH1*, 5-ACCACAGTCCATGCCATCAC-3 (F), 5-TCCACCACCCTGTTGCTGTA-3 (R) for *GAPDH*. The reaction conditions were 35 cycles at 94°C for 30 sec, Tm for 30 sec, and 72°C for 30 sec. *GAPDH* was used as an internal loading control. Real-time quantitative PCR was carried out in a Roche LightCycler480 Real-Time PCR System (Roche) using SYBR Green detection system (Bio-rad, Hercules, CA, USA). The relative level of gene expression was calculated using the cycle threshold (*C*_*t*_) method. The mean *C*_*t*_ values from duplicate measurements were used to calculate the expression of the target gene using the following formula: fold change in gene expression, 2^−ΔΔ*C*_*t*_^ = 2^−(Δ*C*_*t*_  (treated  samples)−Δ*C*_*t*_  (untreated  control))^, where Δ*C*_*t*_ = *C*_*t*_  (Gene  of  interest) − *C*_*t*_  (GAPDH).

### 2.7. Statistical Analyses

All the experiments were repeated independently at least three times. Statistical significance among treatments was evaluated using one-way ANOVA followed by Tukey test. *P* < 0.05 was considered significant.

## 3. Results

### 3.1. EGCG and SFN Can Inhibit Proliferation, Induce Apoptosis and Enhance Efficacy of Cisplatin in Ovarian Cancer Cells

**Figure 1:**
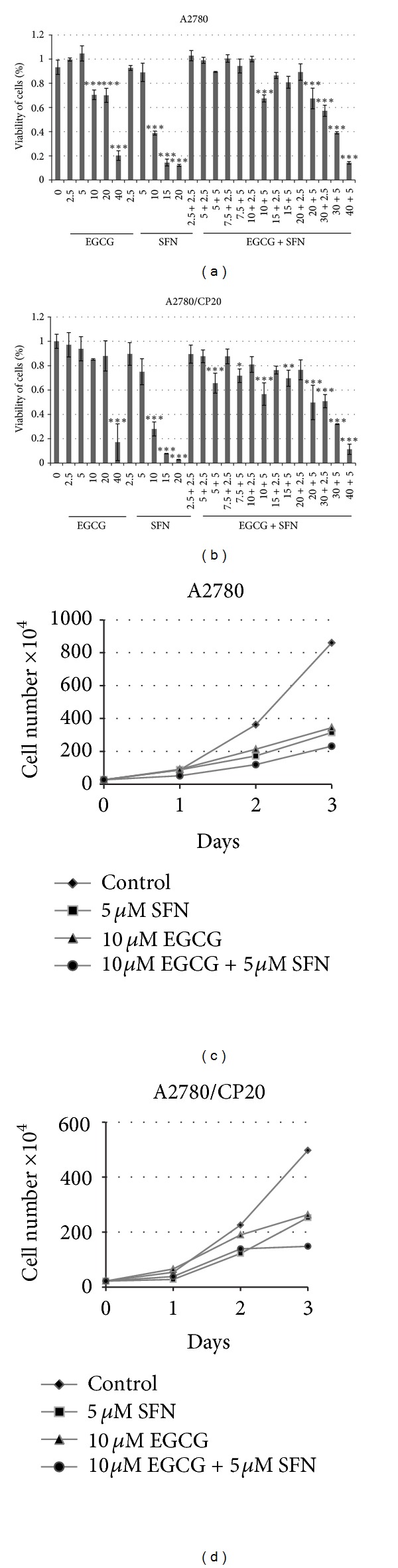
Effect of EGCG and SFN on cisplatin-sensitive (A2780) and cisplatin-resistant (A2780/CP20) ovarian cancer cells. (a) and (b) EGCG and SFN dose-dependently inhibit viability of cisplatin-sensitive (A2780) and cisplatin-resistant ovarian cancer cells (A2780/CP20). EGCG enhances the SFN-mediated growth inhibiting effect in both cell lines. Cells were treated with 2.5, 5, 10, 20, and 40 *μ*M of EGCG, 2.5, 5, 10, 15, and 20 *μ*M of SFN or combination of EGCG and SFN (the first number represents the concentration of EGCG used, while the second number represents the concentration of SFN used). (c) and (d) EGCG and SFN time-dependently inhibit proliferation of cisplatin-sensitive (A2780) and cisplatin-resistant (A2780/CP20) ovarian cancer cells. *P < 0.05, **P < 0.01, ***P < 0.001.

To study the effect of EGCG and SFN treatment on the viability of ovarian cancer cells, we first performed MTT assays to evaluate the optimal doses of EGCG and SFN on inhibiting ovarian cancer cells. As shown in Figure 1, human ovarian cancer cells A2780 and A2780/CP20 were treated with the indicated concentrations of EGCG and SFN for 72 h. We observed a dose-dependent cell growth inhibition with EGCG, SFN treatment alone, or EGCG and SFN combinational treatments in both A2780 and A2780/CP20 cells. Furthermore, ovarian cancer cells were treated with indicated doses for 24, 48, and 72 h, and we observed a time-dependent inhibition on growth compared with control. Combination treatment of 10 *μ*M EGCG and 5 *μ*M SFN exhibits stronger inhibiting effect compared with 5 *μ*M SFN alone in cisplatin-resistant A2780/CP20 ovarian cancer cells.

**Figure 2:**
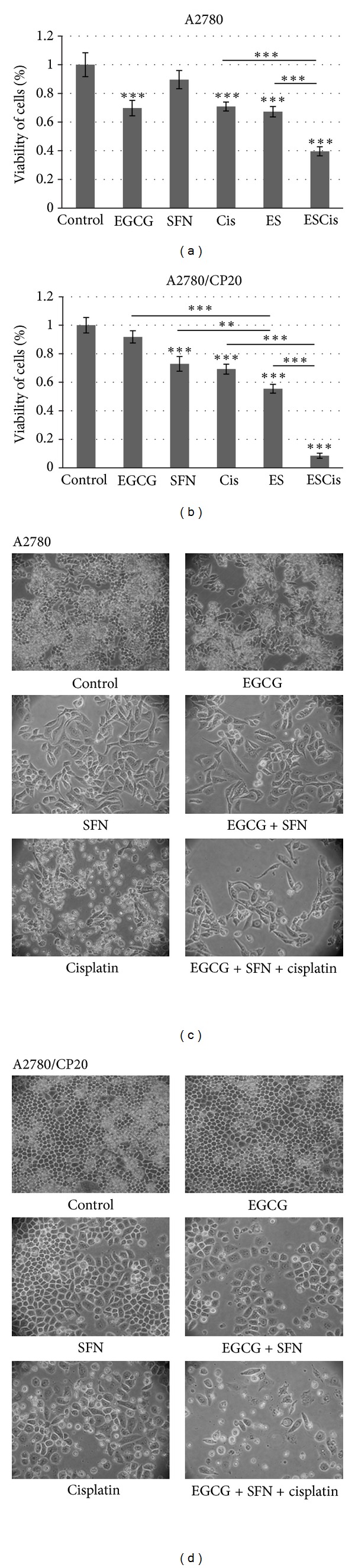
Effect of EGCG and SFN on the efficacy of cisplatin to cisplatin-sensitive (A2780) and cisplatin-resistant (A2780/CP20) ovarian cancer cells. (a) and (b). EGCG and SFN combinational treatment can enhance the efficacy of cisplatin in both cisplatin-sensitive and cisplatin-resistant ovarian cancer cells. (c) and (d) Morphology of ovarian cancer cells after 3 days of EGCG, SFN, or cisplatin treatment. EGCG: 10 *μ*M, SFN: 5 *μ*M, ES: EGCG 10 *μ*M + SFN 5 *μ*M, Cis: cisplatin, 1.5 *μ*M cisplatin for A2780, 8 *μ*M cisplatin for A2780/CP20, ESCis: EGCG 10 *μ*M + SFN 5 *μ*M + cisplatin (1.5 *μ*M cisplatin for A2780, 8 *μ*M cisplatin for A2780/CP20). Magnification: 100X. *P < 0.05, **P < 0.01, ***P < 0.001.

The morphology of human ovarian cancer cells were further studied with 10 *μ*M EGCG and 5 *μ*M SFN combination treatment. As shown in Figure 2, SFN treatments clearly inhibited cellular proliferation in these ovarian cancer cells. In addition, EGCG and SFN combination treatments have an even stronger inhibiting effect on cisplatin-resistant (A2780/CP20) ovarian cancer cells compared with each compound administered singly.

Furthermore, cisplatin was incorporated in the study to examine the effect of EGCG and SFN combinational treatment on the efficacy of cisplatin to ovarian cancer cells. Increasing doses of cisplatin were applied to both cisplatin-sensitive (A2780) and cisplatin-resistant (A2780/CP20) ovarian cancer cells to find the effective dose for further study. Doses that kill approximately 70% of cells by 3 days in each cell line (1.5 *μ*M for A2780 and 8 *μ*M for A2780/CP20, data not shown) were further used for combinational study. EGCG, SFN and cisplatin were then applied to cisplatin-sensitive and cisplatin-resistant ovarian cancer cells. As shown in Figures 2(c) and 2(d), EGCG and SFN combination treatment can enhance efficacy of cisplatin to both ovarian cancer cell types.

**Figure 3:**
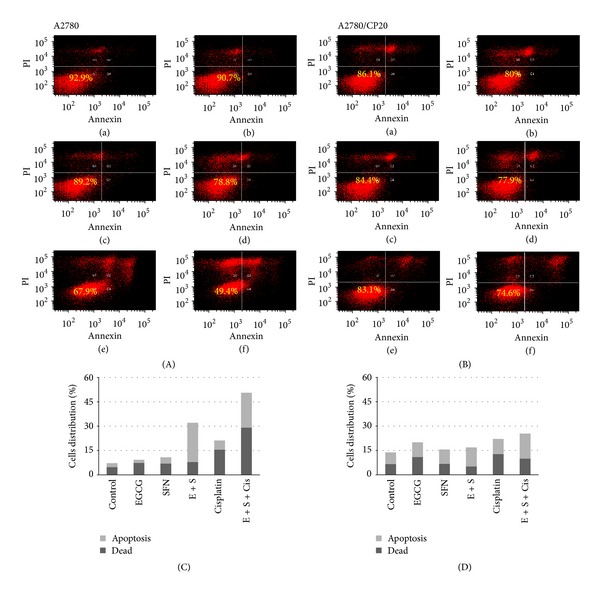
EGCG and SFN combination treatment can induce apoptosis of ovarian cancer cells and enhance the apoptosis-induction effect of cisplatin. (A) and (C) Apoptosis induced by EGCG, SFN, and cisplatin treatment in A2780. (B) and (D) Apoptosis induced by EGCG, SFN and cisplatin treatment in A2780/CP20. Apoptosis cells include both early apoptosis and late apoptosis which are corresponding to right lower and upper quadrants. Dead cells correspond to the left upper quadrant. (a) control; (b) 10 *μ*M EGCG; (c) 5 *μ*M SFN; (d) 10 *μ*M EGCG + 5 *μ*M SFN; (e) 1.5 *μ*M cisplatin for A2780, 8 *μ*M cisplatin for A2780/CP20; (f) 10 *μ*M EGCG + 5 *μ*M SFN + 1.5 *μ*M cisplatin for A2780, 10 *μ*M EGCG + 5 *μ*M SFN + 8 *μ*M cisplatin for A2780/CP20. A2780 and A2780/CP20 were treated with EGCG or SFN for 3 days.

We next performed apoptosis analysis of both cell types in response to treatments with EGCG, SFN and cisplatin to reveal potential mechanisms for the inhibition. As shown in Figure 3, EGCG and SFN combination treatment induced more cells to undergo apoptosis in A2780 cells as compared with EGCG or SFN alone, while EGCG, SFN, and cisplatin cotreatment induced even more cells to undergo apoptosis. However, this apoptosis-induction effect is not obvious in A2780/CP20 cells, suggesting that other mechanisms are involved in the inhibiting effect of EGCG, SFN, and cisplatin co-treatment in this resistant cell type.

### 3.2. EGCG and SFN Combination Treatment Can Enhance Cisplatin-Mediated G2/M Phase Arrest

**Figure 4:**
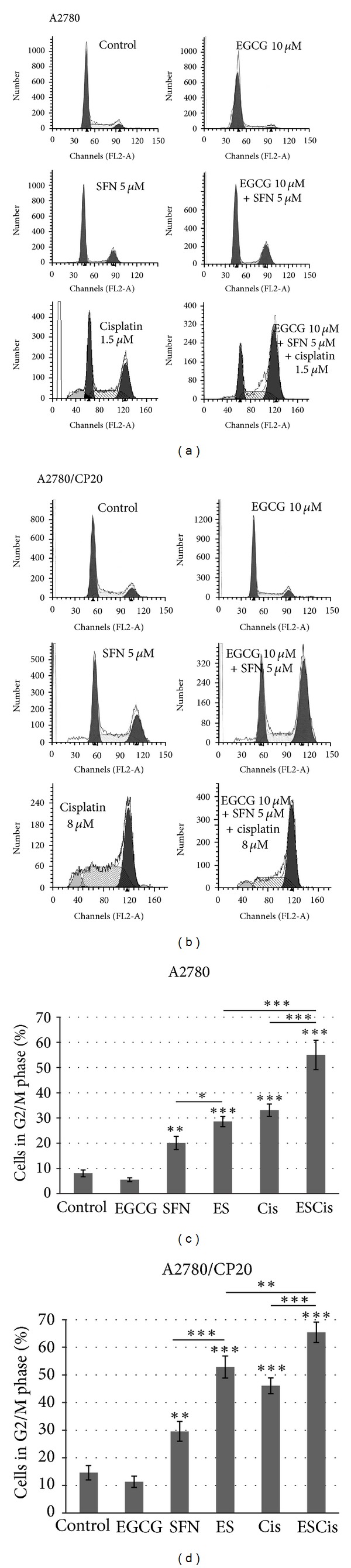
EGCG and SFN combination treatment enhances cisplatin-mediated G2/M phase arrest. SFN arrests the cell cycle at G2/M phase in ovarian cells, and EGCG potentiates the G2/M phase arrest of SFN. A2780 or A2780/CP20 cells were treated with 10 *μ*M EGCG or 5 *μ*M SFN or 1.5 *μ*M (for A2780)/8 *μ*M (for A2780/CP20) cisplatin for 3 days. All the experiments were repeated at least 3 times. ES: EGCG + SFN, Cis: cisplatin, ESCis: EGCG + SFN + cisplatin. *P < 0.05, **P < 0.01, ***P < 0.001.

To further reveal the mechanisms behind the proliferation inhibition effect of EGCG and SFN on ovarian cancer cells, cell cycle progression with EGCG, SFN, and cisplatin treatment was analyzed. As shown in Figure 4, cisplatin can arrest both A2780 and A2780/CP20 cells in G2/M phase which is consistent with previous reports from other research groups [20, 21]. EGCG has no obvious effect on cell cycle progression at 10 *μ*M compared with control, while SFN at 5 *μ*M can arrest both A2780 and A2780/CP20 in G2/M phase. The combination of EGCG and SFN treatments leads to even more cells arrested in G2/M phase compared with SFN treatment alone in both A2780 and A2780/CP20, suggesting that EGCG can potentiate the SFN-mediated G2/M arresting effect. Cotreatment with EGCG and SFN and cisplatin leads to more cells arrested in G2/M phase compared with EGCG and SFN combination treatment alone.

### 3.3. **p*21* mRNA and Protein Expression Are Upregulated in Cisplatin-Sensitive (A2780) Ovarian Cancer Cells

**Figure 5:**
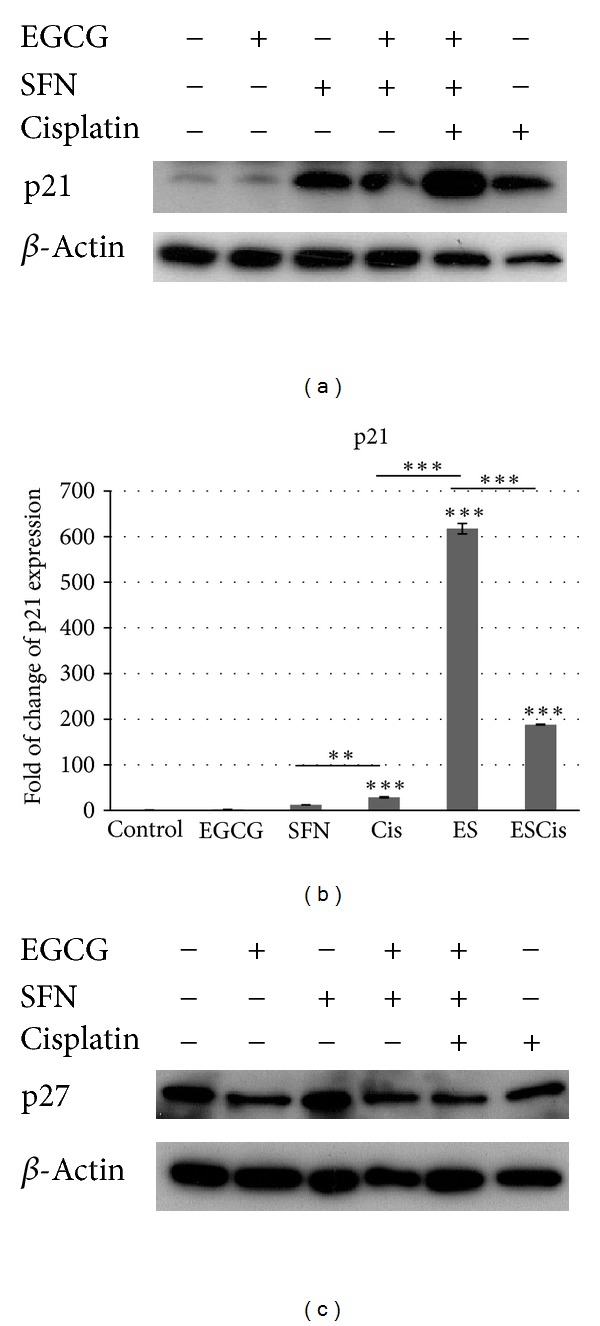
EGCG and SFN combinational treatment can enhance p21 level induced by cisplatin in A2780 ovarian cancer cells. (a) Cisplatin can induce p21 protein expression in A2780 cells, while EGCG and SFN combinational treatment induces an even higher level of p21. (b) EGCG and SFN combination treatment can induce a higher level of *p21* mRNA than either compound alone as evaluated by real-time PCR. (c) Expression level of p27 was not regulated by EGCG, SFN, or cisplatin treatment. All the experiments were repeated at least 3 times. ES: EGCG + SFN, Cis: cisplatin, ESCis: EGCG + SFN + cisplatin. *P < 0.05, **P < 0.01, ***P < 0.001.

To reveal molecular mechanisms of the G2/M phase arrest of ovarian cancer cells mediated by EGCG and SFN combinational treatment, expression levels of cell cycle inhibitors were analyzed through both western blot studies and real-time quantitative PCR. As shown in Figure 5, p21 was upregulated by SFN or EGCG and SFN combination treatment in A2780 cells. Real-time PCR indicates that *p21* was upregulated by SFN treatment alone, while EGCG can potentiate the upregulation of *p21* mediated by SFN treatment. Other CDKIs (cyclin-dependent kinase inhibitors) such as p27 were also evaluated and no alteration on p27 expression was detected (Figure 5(c)). The expression of a frequently silenced tumor suppressor gene (hMLH1) in cisplatin-resistant ovarian cancer cells was also examined in EGCG and SFN treatments. Although we detected upregulation of *hMLH1* mRNA level in A2780/CP20 cells, we did not detect changes in hMLH1 protein expression (data not shown).

## 4. Discussion

We determined the effect of EGCG and SFN combination treatment on both cisplatin-sensitive and cisplatin-resistant ovarian cancer cells and the effect on the efficacy of cisplatin to these cancer cells to elucidate the potential mechanisms responsible for the effects. We used cisplatin-sensitive A2780 and cisplatin-resistant A2780/CP20 ovarian cancer cells as a model. The A2780 cells were established from tumor tissue derived from an untreated patient. By contrast, the A2780/CP20 cells were developed by sequential exposure of the A2780 cell line to increasing concentrations of cisplatin. A2780 and A2780/CP20 cells have been widely used as a cell model to study the cisplatin resistance of ovarian cancer [22–24]. Our findings indicate that SFN inhibits both cisplatin-sensitive and cisplatin-resistant ovarian cancer cells while EGCG enhances the inhibiting effect of SFN. Furthermore, we found that EGCG and SFN combination treatments enhance the efficacy of cisplatin in both cisplatin-sensitive and cisplatin-resistant ovarian cancer cells. Mechanistic studies reveal that EGCG and SFN combination treatments upregulate p21 expression, while EGCG, SFN, and cisplatin treatment induces even higher levels of p21 compared with EGCG and SFN combinational treatment alone. These findings reveal for the first time that EGCG and SFN combination treatments inhibit proliferation of ovarian cancer cells and enhance the efficacy of cisplatin on ovarian cancer cells by upregulating p21.

Progression through each phase of the cell cycle is regulated carefully to avoid proliferation or mitosis when adverse conditions exist. Cells can be arrested in G1, S, and G2/M phases to prevent replication of damaged DNA or to prevent aberrant mitosis. Our results indicate that SFN treatment arrests ovarian cancer cells in G2/M phase, while EGCG enhances SFN-mediated G2/M phase arrest. Apoptosis analyses revealed that EGCG enhances SFN-mediated apoptosis in both cisplatin-sensitive and cisplatin-resistant ovarian cancer cells. Furthermore, EGCG and SFN combination treatments promote apoptosis induced by cisplatin in both cell types accompanied by more cells arrested in G2/M phase. These results indicate that EGCG and SFN combination treatments exert anticancer effects by inhibiting cell cycle progression at G2/M phase and induction of apoptosis in both cisplatin-sensitive and cisplatin-resistant ovarian cancer cells. EGCG and SFN combinational treatment also promotes cisplatin-mediated apoptosis by enhancing more cells arrested in G2/M phase.

p21 is known as a cell cycle inhibitor involved in G2/M phase progression. Its upregulation has been linked to cell cycle arrest at G1 or G2/M phase which may contribute to the proliferation inhibiting effect of a number of dietary compounds in cancer cells. In this study, EGCG was found to enhance SFN-mediated upregulation of p21 mRNA and protein expression in A2780 cells. However, this upregulation of p21 was not detected in A2780/CP20 cells which may contribute to the different responses of A2780 and A2780/CP20 cells to EGCG and SFN combination treatments. This reflects the complex regulation of p21 in ovarian cancer cells.

We also assessed the effect of EGCG and SFN treatment on the expression of *hMLH1 *gene. *hMLH1* is involved in the pathway that relates DNA damage signals caused by cytotoxic chemotherapies such as cisplatin treatment to cellular apoptosis machinery. Silencing of its expression or loss of function of the protein has been associated with resistance to chemotherapy in ovarian cancer cells. Furthermore, a previous study indicates that EGCG can induce its expression in breast cancer cell lines [25]. Thus, we had proposed that EGCG and SFN treatment may increase the efficacy of cisplatin on cisplatincisplatin-resistant ovarian cancer cells through upregulating *hMLH1* expression. Although we detect upregulation of *hMLH1* mRNA in A2780/CP20 cells, we did not detect its expression at the protein level, suggesting that hMLH1 is not crucial to the effect of EGCG and SFN treatment which we observed in ovarian cancer cells. 

The dose of EGCG and SFN used in this experiment is physiologically achievable in humans through consumption of green tea and broccoli sprouts. Generally, consumption of a few cups (~3-4) of green tea and one cup of broccoli sprouts can reach the effective doses of EGCG and SFN in this study. 

EGCG and SFN are also known for their antioxidative effects [26, 27]. Currently, concurrent use of antioxidants with conventional chemotherapy in cancer patients is still controversial. On one side, the argument is that conventional chemotherapies take advantage of reactive oxygen species (ROS) to kill cancer cells, while antioxidants will scavenge ROS and thus attenuate the efficacy of chemotherapies [28]. On the other side, the argument is that chemotherapy depends on other mechanisms instead of ROS to kill cancer cells, and ROS is actually responsible for side effects of chemotherapy and also interferes with chemotherapy. Thus, scavenging ROS through applying oxidants can actually enhance the toxicity of chemotherapy and minimize its side effects [29]. For cisplatin-based chemotherapy, cisplatin in clinical doses kills cancer cells significantly through incorporating into DNA double-strands and causing DNA damage signals to induce apoptosis which is not dependent on ROS-mediated cell death [30]. This suggests that the ROS scavenging effect of antioxidants will not attenuate efficacy of cisplatin against cancer cells. The present study clearly demonstrates that EGCG and SFN combinational treatment can enhance the efficacy of cisplatin in both cisplatin-sensitive and cisplatin-resistant ovarian cancer cells. Furthermore, our results indicate that EGCG and SFN combinational treatment can upregulate p21 and arrest cells in G2/M phase. However, further animal and clinical studies are warranted to illustrate whether EGCG and SFN combinational treatments can enhance the efficacy of cisplatin-based chemotherapy *in vivo* and in ovarian cancer patients.

## 5. Conclusion

In conclusion, our data show for the first time that EGCG can potentiate the G2/M phase arrest of SFN to inhibit proliferation of ovarian cancer cells and induce apoptosis of these cells. Furthermore, EGCG and SFN combination treatments enhance the efficacy of cisplatin in both cisplatin-sensitive and cisplatin-resistant ovarian cancer cells. These results suggest the potential of applying EGCG and SFN combination treatment for assisting conventional platinum/taxane combination-based chemotherapy as a novel anticancer approach.
